# ﻿Testing conflicting taxonomic hypotheses in myrmecophilous *Oochrotus* Lucas, 1852 (Coleoptera, Tenebrionidae)

**DOI:** 10.3897/zookeys.1258.155620

**Published:** 2025-11-03

**Authors:** Julene Gómez-Vicioso, Álvaro Conca-Esquembre, Pilar Jurado-Angulo, Mario García-París

**Affiliations:** 1 Department of Biodiversity and Evolutionary Biology, Museo Nacional de Ciencias Naturales (MNCN-CSIC), c/ José Gutiérrez Abascal, 2. 28006, Madrid, Spain Museo Nacional de Ciencias Naturales Madrid Spain; 2 Division of Ecology and Evolution (E&E), Research School of Biology (RSB), Australian National University, Canberra, ACT, Australia Australian National University Canberra Australia; 3 CIBIO, Centro de Investigação em Biodiversidade e Recursos Genéticos, InBIO Laboratório Associado e Faculdade de Ciências da Universidade do Porto, Vairão, Portugal InBIO Laboratório Associado e Faculdade de Ciências da Universidade do Porto Vairão Portugal; 4 Universidade Técnica do Atlântico, UTA – Instituto de Engenharias e Ciências do Mar (ISECMAR), Mindelo, Cabo Verde Universidade Técnica do Atlântico Mindelo Cabo Verde

**Keywords:** Beetles, evolution, mitochondrial DNA, molecular analysis, nuclear DNA, phylogeography, western Mediterranean

## Abstract

Cryptic and pseudocryptic species are common in myrmecophilous insects, making their taxonomic classification complex when based solely on morphology. This is the case for the beetles of the genus *Oochrotus* Lucas, 1852, a group of small tenebrionids inhabiting ant nests. In 1961, Canzoneri described one new species and eight subspecies based on the morphology of the aedeagus and ovipositor. However, in 2000, Soldati and Soldati synonymised most of these taxa, arguing that the differences found by Canzoneri were not significant. The aim of our study was to test these two competing hypotheses using a molecular approach. For this purpose, partial sequences of the nuclear gene ITS2 and the mitochondrial gene *cytb* were obtained from individuals from North Africa, Italy, and the Iberian Peninsula, followed by phylogenetic analyses based on Bayesian inference. The results show that specimens from these three territories are in separate lineages corresponding to three different species: 1) *O. unicolor* Lucas, 1852; 2) *O. laurae* Canzoneri, 1961, **stat. rev.**, and 3) *O. lusitanicus* Canzoneri, 1961, **stat. nov.** (= *O. u. espagnoli* Canzoneri, 1961, **syn. nov.**; = *O. u. hispanus* Canzoneri, 1961, **syn. nov**.; = *O. u. meridionalis* Canzoneri, 1961, **syn. nov.**). This new proposal diverges from both preceding hypotheses, showing an intermediate level of diversity between the two. This reflects that species of the genus *Oochrotus* are probably pseudocryptic, whose morphological examination may lead to misidentification in the absence of molecular data.

## ﻿Introduction

Myrmecophilous species are those species that live in association with ants. The term myrmecophilous encompasses a total of about 10,000 species of arthropods (Elmes 1996), although not all of them are related to ants in the same way. The classical concept involves a mutualistic type of relationship, usually found in the order Hemiptera (Ivens, 2015). However, other insects have a parasitoid or social parasitism association with ants. The latter case occurs in the order Coleoptera, in which beetles exploit the resources and social structure of the ants, inflicting a cost on their communities, but without increasing direct mortality (Parker 2016).

Myrmecophilous beetles are currently known to occur in at least 33 families within the order Coleoptera (Hölldobler and Wilson 1990; Parker 2016). In general, this interaction has been most studied in the subfamilies Aleocharinae (Kistner 1993) and Pselaphinae (Chandler 2001) within Staphylinidae, in the subfamily Paussinae (Geiselhardt et al. 2007) within the family Carabidae (Di Giulio et al. 2003), and in some species of the family Coccinellidae (Vantaux et al. 2012) and Scarabaeidae (Vaz-de-Mello et al. 1998). Through the study of the different lineages, it has been observed that myrmecophilous beetles tend to have common adaptations that allow them to access ant nests, integrate into colonies and exploit their resources. These include, among others, the ability to camouflage themselves chemically (Vander Meer and Wojcik 1982; Akino 2002; Lenoir et al. 2013) and to present morphological structures that make them difficult for ants to attack (Parker 2016). In addition to converging on similar morphological features, these beetles tend to share common traits across closely related species, a pattern also observed in other groups of myrmecophilous insects (Thomas et al. 1989; Elmes et al. 1994, 1999; Schönrogge et al. 2002). This morphological homogeneity is a source of conflict for taxonomic studies, often rendering the definition of taxa complicated.

One example of taxonomic complexity is provided by the genus *Oochrotus* Lucas, 1852. *Oochrotus* is a genus of small myrmecophilous tenebrionid beetles included within the tribe Crypticini in the subfamily Diaperinae (Iwan et al. 2020). They are small organisms (2–3 mm), with an ovoid convex body. As regards their colouring, they present an earthy orange-brown testaceous colour, somewhat similar to that of the arid substrates they inhabit. It is noteworthy that they lack posterior wings and that they have no eyes, the latter character being used to separate them from the rest of the Palaearctic Crypticini (Español 1955). Their distribution is limited to the Mediterranean region (Canzoneri 1961; Español 1963; Cabon and Soldati 2024), where they are found inhabiting the nests of the ants of the genus *Messor* Forel, 1890 (Español 1949, 1955, 1963). They feed on the remains of seeds, flour, or other debris in the waste pits of the ants’ nests (Parmentier et al. 2019).

The taxonomy of the genus *Oochrotus* is still unresolved, and the authors who have worked with the group have presented very different perspectives on the internal diversity within it. After the description of the genus *Oochrotus* based on its type species, *O. unicolor* Lucas, 1852, little further work was done on the diversity of the group during the next century, the most notable being the description of the Eastern Mediterranean *O. glaber* Demaison, 1905 (Lucas 1852; Demaison 1905; Lokay 1907; Koch 1935). It was not until Canzoneri (1961) that an intensive effort was made to describe the diversity within the genus, based mainly on the morphology of the aedeagus and the distal end of the ovipositor. Canzoneri (1961) retained *O. glaber* and *O. unicolor*, and defined six additional subspecies within *O. unicolor*, four of them distributed in different regions of the Iberian Peninsula, and two in Italy. He also described a new species, *O. laurae* Canzoneri, 1961, with two subspecies distributed in Italy. Subsequently, Rallo (1974) disregarded *O. laurae*, including its two subspecies within *O. unicolor*, and he also described a new Italian subspecies. Soldati and Soldati (2000) presented a totally different perspective, and after reviewing several populations of *O. unicolor*, they considered that the morphological differences reported by Canzoneri (1961) were not significant, synonymising six subspecies with *O. u. unicolor*. Soldati and Soldati (2000) additionally remarked that, despite their inability to study all the subspecies proposed by Canzoneri (1961), future revisionary efforts will most likely be able to generalize the synonymy of all the remaining subspecies.

The aim of our study was to test the conflicting hypotheses proposed by Canzoneri (1961) and Soldati and Soldati (2000) with respect to the internal subdivision of *O. unicolor* by using a molecular approach. The first hypothesis suggests that two species with nine subspecies can be distinguished within the group, whereas the second considers one species including four not studied subspecies (see Table 1 for details). These contrasting hypotheses are based on the relative importance given to certain morphological characters as taxonomically diagnostic, more particularly to the genital structures. To test them, we performed phylogenetic analyses with partial sequences of two widely used DNA markers, mtDNA cytochrome b gene (*cytb*) and nuclear internal transcribed spacer 2 (ITS2) and revised previous statements of morphological diversification.

**Table 1.** Taxonomic hypotheses proposed by Canzoneri (1961), Soldati and Soldati (2000) and the present study with respect to the internal subdivision of *Oochrotus unicolor.* The different taxonomic positions of the described (sub)species are shown according to each proposal, together with their type localities. Taxa regarded as valid under each hypothesis are shown in **bold** and, when synonymised, the taxon in which they are included is shown in regular font. Taxonomic decisions newly proposed in this study are indicated with light gray shading. Taxa not examined in this study and whose taxonomic assignments were based on Canzoneri (1961) or Soldati and Soldati (2000) are marked with an asterisk (*). Note that *O. unicolor chilivanii* does not appear in Canzoneri’s original hypothesis (indicated by ‘–’ in the table), as it was described later by Rallo (1974); it is nevertheless included here for clarity.

Canzoneri (1961)	Soldati and Soldati (2000)	This study	Type locality
***O. laurae laurae*** Canzoneri, 1961	*O. unicolor unicolor*	***O. laurae* stat. rev.**	“Moscona (Grossetto)”
***O. laurae sardous*** Canzoneri, 1961	*O. unicolor unicolor*	O. unicolor unicolor*	“Flumentorgiu (Sardegna)”
***O. unicolor unicolor*** Lucas, 1852	***O. unicolor unicolor***	***O. unicolor unicolor***	“plateaux de Médéah et de Boghar”
***O. unicolor ardoini*** Canzoneri, 1961	***O. unicolor ardoini***	O. unicolor ardoini*	“Roma dintorni”
***O. unicolor chilivanii*** Rallo, 1974 (–)	*O. unicolor unicolor*	O. unicolor unicolor*	“Chilivani (Sassari)”
***O. unicolor espagnoli*** Canzoneri, 1961	*O. unicolor unicolor*	*O. lusitanicus* **syn. nov.**	“Tiana, prov. Barcellona”
***O. unicolor hispanus*** Canzoneri, 1961	*O. unicolor unicolor*	*O. lusitanicus* **syn. nov.**	“Robledo (Madrid)”
***O. unicolor lusitanicus*** Canzoneri, 1961	***O. unicolor lusitanicus***	***O. lusitanicus* stat. nov.**	“Evora (Portogallo)”
***O. unicolor meridionalis*** Canzoneri, 1961	*O. unicolor unicolor*	*O. lusitanicus* **syn. nov.**	“Algeciras”
***O. unicolor moltonii*** Canzoneri, 1961	***O. unicolor moltonii***	O. unicolor moltonii*	“Ficuzza (Palermo)”

## ﻿Methods

### ﻿Taxon sampling

Specimens were collected opportunistically at 10 localities in the Iberian Peninsula, North Africa, and Italy with the aim of covering part of the known distribution of the group. Sampling was initially directed towards type localities, but most of the specimens were found in non-selected areas. Identification was primarily based on geography, with a review of morphological characters, especially in the case of the Italian population (see Results). Specimens were located under stones, along tunnels occupied by ants of the genus *Messor* or walking on the underside of the stones, generally in grassland areas, at the edge of *Quercus rotundifolia* Lam. patches. At each locality individuals were visually searched, hand-collected, and georeferenced. All were preserved in 96% to absolute ethanol. In each population, 1–15 individuals were included in the molecular analysis, and at least one individual was retained for future morphological studies. All specimens are stored at the Museo Nacional de Ciencias Naturales (**MNCN-CSIC**) (Madrid, Spain).

### ﻿DNA extraction and sequencing

DNA was extracted from a total of 57 individuals of the genus *Oochrotus* (Table 2). For this purpose, the specimens were punctured in the upper abdomen and the entire individuals were included in the extraction buffer. Total genomic DNA was extracted using the Qiagen DNeasy extraction kit (Qiagen) and following the protocol indicated by the manufacturer.

**Table 2.** List of *Oochrotus* specimens included in the study. Species identity, collection localities and coordinates, voucher numbers, MNCN-CSIC identification codes and GenBank accession numbers are shown.

Species	Locality	GPS Coordinates	Voucher	MNCN	Genbank *cytb*	Genbank ITS2
*O. laurae*	Lazio: Ciudad metropolitana de Roma Capital: Allumiere	42°08'00"N; 11°54'07"E	jgv22001a	366745	PQ376647	PQ348543
	Lazio: Ciudad metropolitana de Roma Capital: Allumiere	42°08'00"N; 11°54'07"E	jgv22002a	366746	PQ376648	
	Lazio: Ciudad metropolitana de Roma Capital: Allumiere	42°08'00"N; 11°54'07"E	jgv22003a	366747	PQ376649	PQ348544
	Lazio: Ciudad metropolitana de Roma Capital: Allumiere	42°08'00"N; 11°54'07"E	jgv22005a	389968	PQ376650	PQ348545
*O. lusitanicus*	Andalucía: Huelva: Santa Olalla del Cala	37°54'25"N; 6°14'09"W	jgv22007b	389969	PQ376651	PQ348546
*O. lusitanicus*	Andalucía: Huelva: Santa Olalla del Cala	37°54'25"N; 6°14'09"W	jgv22008b	389970	PQ376652	PQ348547
*O. lusitanicus*	Andalucía: Huelva: Santa Olalla del Cala	37°54'25"N; 6°14'09"W	jgv22009b	389971	PQ376653	PQ348548
*O. lusitanicus*	Andalucía: Huelva: Santa Olalla del Cala	37°54'25"N; 6°14'09"W	jgv22010b	389972	PQ376654	PQ348549
*O. lusitanicus*	Andalucía: Huelva: Santa Olalla del Cala	37°54'25"N; 6°14'09"W	jgv22011b	389973	PQ376655	PQ348550
*O. lusitanicus*	Andalucía: Sevilla: El Coronil	37°03'02"N; 5°37'40"W	jgv22064j	390007	PQ376681	PQ348584
*O. lusitanicus*	Andalucía: Sevilla: El Coronil	37°03'02"N; 5°37'40"W	jgv22065j	390008	PQ376682	PQ348585
*O. lusitanicus*	Andalucía: Sevilla: El Coronil	37°03'02"N; 5°37'40"W	jgv22066j	390009	PQ376683	PQ348586
*O. lusitanicus*	Andalucía: Sevilla: El Coronil	37°03'02"N; 5°37'40"W	jgv22067j	390010	PQ376684	
*O. lusitanicus*	Andalucía: Sevilla: El Coronil	37°03'02"N; 5°37'40"W	jgv22068j	390011		PQ348587
*O. lusitanicus*	Andalucía: Sevilla: El Coronil	37°03'02"N; 5°37'40"W	jgv22069j	390012	PQ376685	PQ348588
*O. lusitanicus*	Andalucía: Sevilla: El Coronil	37°03'02"N; 5°37'40"W	jgv22071j	390013		PQ348589
*O. lusitanicus*	Andalucía: Sevilla: El Coronil	37°03'02"N; 5°37'40"W	jgv22072j	390014	PQ376686	PQ348590
*O. lusitanicus*	Andalucía: Sevilla: El Coronil	37°03'02"N; 5°37'40"W	jgv22073j	390015	PQ376687	
*O. lusitanicus*	Andalucía: Sevilla: El Coronil	37°03'02"N; 5°37'40"W	jgv22074j	390016		PQ348591
*O. lusitanicus*	Andalucía: Sevilla: El Coronil	37°03'02"N; 5°37'40"W	jgv22075j	390017	PQ376688	PQ348592
*O. lusitanicus*	Andalucía: Sevilla: El Coronil	37°03'02"N; 5°37'40"W	jgv22076j	390018	PQ376689	PQ348593
*O. lusitanicus*	Andalucía: Sevilla: El Coronil	37°03'02"N; 5°37'40"W	jgv22077j	390019	PQ376690	PQ348594
*O. lusitanicus*	Andalucía: Sevilla: El Coronil	37°03'02"N; 5°37'40"W	jgv22078j	390020		PQ348595
*O. lusitanicus*	Andalucía: Sevilla: El Coronil	37°03'02"N; 5°37'40"W	jgv22079j	390021	PQ376691	PQ348596
*O. lusitanicus*	Castilla-La Mancha: Albacete: Chinchilla de Montearagón	38°55'23"N; 1°43'43"W	jgv22013c	389974	PQ376656	PQ348551
*O. lusitanicus*	Castilla-La Mancha: Albacete: Chinchilla de Montearagón	38°55'23"N; 1°43'43"W	jgv22014c	389975		PQ348552
*O. lusitanicus*	Castilla-La Mancha: Albacete: Chinchilla de Montearagón	38°55'23"N; 1°43'43"W	jgv22015c	389976	PQ376657	PQ348553
*O. lusitanicus*	Castilla-La Mancha: Albacete: Chinchilla de Montearagón	38°55'23"N; 1°43'43"W	jgv22016c	389977	PQ376658	PQ348554
*O. lusitanicus*	Castilla-La Mancha: Albacete: Chinchilla de Montearagón	38°55'23"N; 1°43'43"W	jgv22017c	389978	PQ376659	PQ348555
*O. lusitanicus*	Castilla-La Mancha: Albacete: Chinchilla de Montearagón	38°55'23"N; 1°43'43"W	jgv22018c	389979	PQ376660	PQ348556
*O. lusitanicus*	Castilla-La Mancha: Albacete: Chinchilla de Montearagón	38°55'23"N; 1°43'43"W	jgv22019c	389980	PQ376661	PQ348557
*O. lusitanicus*	Castilla-La Mancha: Albacete: Chinchilla de Montearagón	38°55'23"N; 1°43'43"W	jgv22020c	389981	PQ376662	PQ348558
*O. lusitanicus*	Castilla-La Mancha: Albacete: Chinchilla de Montearagón	38°55'23"N; 1°43'43"W	jgv22021c	389982	PQ376663	PQ348559
*O. lusitanicus*	Castilla-La Mancha: Albacete: Chinchilla de Montearagón	38°55'23"N; 1°43'43"W	jgv22022c	389983	PQ376664	PQ348560
*O. lusitanicus*	Castilla-La Mancha: Albacete: Chinchilla de Montearagón	38°55'23"N; 1°43'43"W	jgv22023c	389984	PQ376665	PQ348561
*O. lusitanicus*	Castilla-La Mancha: Ciudad Real: Fontanosas	38°45'48"N; 4°32'34"W	jgv22059i	390003	PQ376677	PQ348580
*O. lusitanicus*	Castilla-La Mancha: Ciudad Real: Fontanosas	38°45'48"N; 4°32'34"W	jgv22060i	390004	PQ376678	PQ348581
*O. lusitanicus*	Castilla-La Mancha: Ciudad Real: Fontanosas	38°45'48"N; 4°32'34"W	jgv22061i	390005	PQ376679	PQ348582
*O. lusitanicus*	Castilla-La Mancha: Ciudad Real: Fontanosas	38°45'48"N; 4°32'34"W	jgv22062i	390006	PQ376680	PQ348583
*O. lusitanicus*	Castilla-La Mancha: Ciudad Real: Poblete	38°54'14"N; 4°00'31"W	jgv22058h	390002	PQ376676	PQ348579
*O. lusitanicus*	Castilla-La Mancha: Toledo: Fuentes	39°40'05"N; 5°03'55"W	jgv22051f	389999	PQ376673	PQ348576
*O. lusitanicus*	Castilla-La Mancha: Toledo: Fuentes	39°40'05"N; 5°03'55"W	jgv22052f	390000	PQ376674	PQ348577
*O. lusitanicus*	Extremadura: Badajoz: Montemolín	38°09'15"N; 6°12'28"W	jgv22057g	390001	PQ376675	PQ348578
*O. lusitanicus*	Extremadura: Badajoz: Villanueva del Fresno	38°22'27"N; 7°09'01"W	jgv22042e	389997		PQ348574
*O. lusitanicus*	Extremadura: Badajoz: Villanueva del Fresno	38°22'27"N; 7°09'01"W	jgv22043e	389998	PQ376672	PQ348575
*O. unicolor*	Tánger-Tetuán-Alhucemas: Fahs-Anyera: Ksar Sghir	35°46'20"N; 5°31'36"W	jgv22026d	389985	PQ376666	PQ348562
*O. unicolor*	Tánger-Tetuán-Alhucemas: Fahs-Anyera: Ksar Sghir	35°46'20"N; 5°31'36"W	jgv22027d	389986	PQ376667	PQ348563
*O. unicolor*	Tánger-Tetuán-Alhucemas: Fahs-Anyera: Ksar Sghir	35°46'20"N; 5°31'36"W	jgv22028d	389987	PQ376668	PQ348565
*O. unicolor*	Tánger-Tetuán-Alhucemas: Fahs-Anyera: Ksar Sghir	35°46'20"N; 5°31'36"W	jgv22029d	389988		PQ348564
*O. unicolor*	Tánger-Tetuán-Alhucemas: Fahs-Anyera: Ksar Sghir	35°46'20"N; 5°31'36"W	jgv22030d	389989	PQ376669	PQ348566
*O. unicolor*	Tánger-Tetuán-Alhucemas: Fahs-Anyera: Ksar Sghir	35°46'20"N; 5°31'36"W	jgv22031d	389990		PQ348567
*O. unicolor*	Tánger-Tetuán-Alhucemas: Fahs-Anyera: Ksar Sghir	35°46'20"N; 5°31'36"W	jgv22032d	389991		PQ348568
*O. unicolor*	Tánger-Tetuán-Alhucemas: Fahs-Anyera: Ksar Sghir	35°46'20"N; 5°31'36"W	jgv22033d	389992	PQ376670	PQ348569
*O. unicolor*	Tánger-Tetuán-Alhucemas: Fahs-Anyera: Ksar Sghir	35°46'20"N; 5°31'36"W	jgv22034d	389993		PQ348570
*O. unicolor*	Tánger-Tetuán-Alhucemas: Fahs-Anyera: Ksar Sghir	35°46'20"N; 5°31'36"W	jgv22035d	389994	PQ376671	PQ348571
*O. unicolor*	Tánger-Tetuán-Alhucemas: Fahs-Anyera: Ksar Sghir	35°46'20"N; 5°31'36"W	jgv22036d	389995		PQ348572
*O. unicolor*	Tánger-Tetuán-Alhucemas: Fahs-Anyera: Ksar Sghir	35°46'20"N; 5°31'36"W	jgv22037d	389996		PQ348573

PCRs were performed to amplify the sequences of the *cytb* and ITS2 markers. For *cytb*, a 25 μl mix was used, which included 17.55 μl of H_2_0, 2.5 μl of Nzytech reaction buffer (10×), 1.75 μl of MgCl_2_ (50 mM), 1 μl of dNTP (10 mM), 0.5 μl of both forward and reverse primers (10 μM), 0.2 μl of Taq polymerase (Nzytech, 5 U/μL) and 1 μl of sample DNA. The primers used were CB-J-10933 (Simon et al. 1994) as forward and CB4 (Pons 2006) as reverse. PCR conditions were as follows: 5 min at 96 °C for initial denaturation, 35–40 cycles of 1 min of denaturation at 94 °C, 1 min of annealing at 40–41 °C and 1 min of extension at 72 °C, with a final extension at 72 °C for 5 min.

In the case of ITS2, a 25 μl mix was used, which included 17.8 μl of H_2_0, 2.5 μl of Nzytech reaction buffer (10×), 1.5 μl of MgCl_2_ (50 mM), 1 μl of dNTP (10 mM), 0.5 μl of both forward and reverse primers (10 μM), 0.2 μl of Taq polymerase (Nzytech, 5 U/μL) and 1 μl of sample DNA. The primers used were Cas5p8sFc as forward and CAS28sB1d as reverse (Ji et al. 2003). PCR conditions were as follows: 5 min at 96 °C for initial denaturation, 40 cycles of 30 s of denaturation at 94 °C, 45 s of annealing at 45 °C and 1 min of extension at 72 °C, with a final extension at 72 °C for 5 min.

The amplification products were verified via electrophoresis on 0.8% agarose gels and then sent for Sanger sequencing to Macrogen Spain Inc. (Macrogen Europe, Amsterdam, The Netherlands). The chromatograms and their sequences were individually checked and then aligned using the ClustalW Multiple Alignment tool (BioEdit Sequence Alignment Editor v. 7.7.1.0).

The sequences of *cytb* for 12 specimens and ITS2 for three specimens were not obtained due to problems in the amplification or sequencing processes. As a result, a dataset of 45 specimens and 349 base pairs for *cytb* and another of 54 specimens and 510 base pairs for ITS2 were obtained.

### ﻿Phylogenetic analysis

Independent phylogenetic analyses were performed for *cytb* and ITS2. For both markers, sequences of phylogenetically close taxa were searched in GenBank Data Libraries and selected as outgroups. For *cytb* a sequence of Strongylium cf. indignum Gebien, 1920 (Accession KX461872.1, Soldati et al. 2016) was used, whereas for ITS2 a sequence of *Tenebrio molitor* Linnaeus, 1758 (Accession AJ635266.1, Bologna et al. 2008) was used.

For both markers, phylogenetic reconstruction was carried out by Bayesian inference using the MrBayes program (Huelsenbeck and Ronquist 2001; Ronquist et al. 2012). Two runs were programmed with four chains that ran over 10 million generations, sampling trees every 100 generations. The program was instructed to calculate and use the best model of substitution and best codon partition scheme. In the case of *cytb* it was the model M_142_ = 111234 and in the case of ITS2 the model M_15_ = 121121 (known as HKY model). The convergence between both chains was evaluated taking into account the maximum-likelihood value. After obtaining all the trees, a consensus tree was generated with all of them, previously eliminating the first 25,000 trees as burn-in.

Phylogeographic analyses were carried out by network reconstruction. For this purpose, allele networks were constructed using Population Analysis with Reticulate Trees (PopART) (Leigh and Bryant 2015), applying the TCS algorithm (Clement et al. 2002). In the occasional cases where nucleotide ambiguity was observed, the allele that was common to the rest of the specimens in the region was selected, following a maximum parsimony criterion.

## ﻿Results

**Figure 1.**
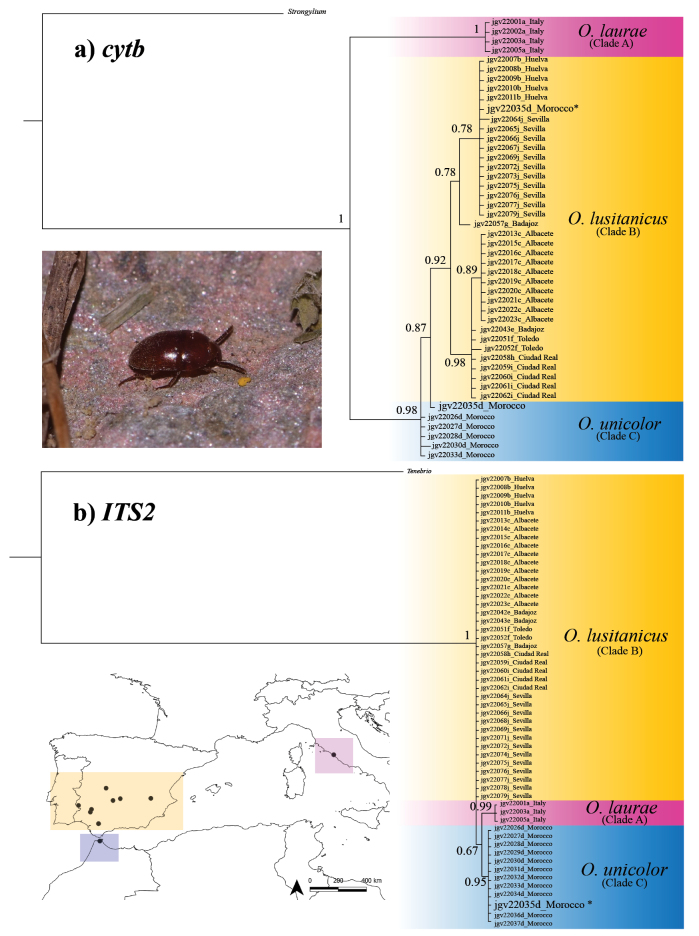
Bayesian phylogenetic hypothesis based on a. *cytb* mitochondrial data and b. ITS2 nuclear data. The colours represent the different lineages recovered in the study and their distribution on the map: *Oochrotus lusitanicus* in the Iberian Peninsula (orange), *O. laurae* in Italy (pink) and *O. unicolor* in North Africa. Numbers near the nodes indicate Posterior Probabilities values (PP). The position in the tree of the alleles of a possible heteroplasmic individual for the *cytb* marker, jgv22035d, is reflected based on two different assumptions. The first is assuming ambiguity at loci with more than one allele (with *) and the second is choosing the most parsimonious alleles according to its population of origin (without *). In both trees there are populations with individuals with identical sequences (the Moroccan in the case of *cytb* and the Iberian Peninsular in the case of ITS2) forming polytomies with phylogenetically close groups. This is an artefact in Bayesian inference when several identical sequences are included in the analyses.

Bayesian reconstruction for *cytb* (Fig. 1a) showed that specimens are clustered in three main groups. Two of them are supported groups, corresponding to the Italian (Clade A, PP = 1) and Spanish (Clade B, PP = 0.92) populations. In the case of the Moroccan population (Clade C), although most individuals are genetically identical, they appear to form a polytomy with the Spanish populations (a common artefact in Bayesian phylogenetic analyses when identical sequences are included, due to the lack of informative sites for resolving branching order). In the case of the ITS2 marker (Fig. 1b), the same three groups are also differentiated. In this case it is the Moroccan (Clade C, PP = 0.95) and Italian (Clade A, PP = 0.99) populations which form well-supported groups, whereas the sequences from the Spanish populations (Clade B) are artefactually represented as a polytomy. The individual jgv22035d from Morocco, which clustered with other Moroccan individuals in the ITS2 tree, showed an ambiguous position in the *cytb* tree. This particular specimen showed a high level of heteroplasmy in the mitochondrial marker *cytb*, producing alleles that cluster in different positions of the tree (Fig. 1).

**Figure 2.**
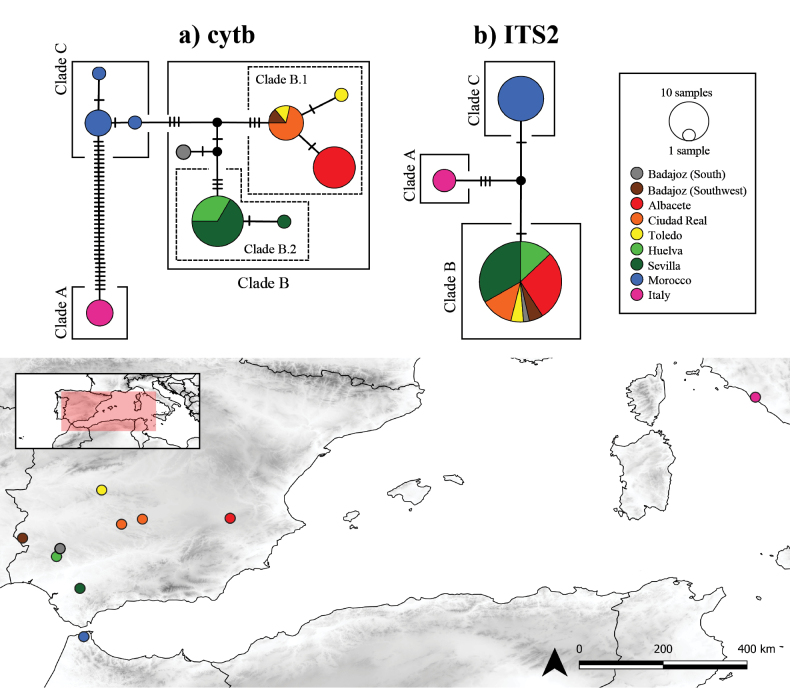
TCS network of *Oochrotus* based on a) the mitochondrial marker *cytb* and b) the nuclear marker ITS2. The size of the circles indicates the relative frequency of sequences belonging to a particular allele and the colours correspond to the geographic origin of the specimens. Inferred intermediate haplotypes are represented by small black circles. The North African, Iberian Peninsula and Italian populations differed from each other in both markers, with no alleles shared between them. In the case of the Iberian Peninsula, some genetic structuring appeared in the *cytb* marker, but it was absent for the ITS2 marker.

The haplotypic network obtained for *cytb* (Fig. 2a) showed a total of 10 haplotypes, separated into three main haplogroups. The first one corresponds to the Italian individuals (Clade A), where a single haplotype is found, clearly differentiated in 35 positions from the next closest haplogroup, the one from Morocco. In Morocco (Clade C), three haplotypes are found, separated by only one position between them. The last haplogroup, that of the Iberian Peninsula (Clade B), is the most complex of them. Here there are a total of six haplotypes, which can be grouped into two subgroups. On the one hand there is a group of specimens from the central-eastern peninsula (Clade B.1), and on the other, those from the southwest (Clade B.2). One haplotype from the south of Badajoz was found in between, close to both groups. It is noteworthy that both subgroups are notably different from each other (seven positions), even more than with respect to the Moroccan population (three and four positions, respectively). In the case of the ITS2 marker (Fig. 2b), three nuclear alleles were found, each corresponding to one of the three main regions studied: Italy (Clade A), the Iberian Peninsula (Clade B), and Morocco (Clade C). The three are closely related, with a difference of two positions between the Iberian Peninsula and Morocco and four positions between these two territories and Italy.

Geographical structuring, and congruence between nuclear and mtDNA markers, support a well-defined taxonomic structure in our sampling reflected in the existence of three evolutionary units, that represent independent species, corresponding to the three main lineages found: North African, Italian, and Iberian. Our sampling is quite representative of the Iberian taxon, that might include all previously proposed names within the region (*O. u. lusitanicus* Canzoneri, 1961; *O. u. espagnoli* Canzoneri, 1961; *O. u. hispanus* Canzoneri, 1961; *O. u. meridionalis* Canzoneri, 1961). Since all the available names were proposed in the same work by Canzoneri (1961), they all have equal priority. We choose as the specific epithet the name with the type locality geographically closest to any of our studied populations (Évora to Villanueva del Fresno), which is *O. lusitanicus* Canzoneri, 1961. The only available name for northwestern Africa is *O. unicolor*, which is therefore retained for the African lineage represented by our Moroccan sample. The Italian name is more problematic since our single sample is almost equally distant from *O. u. ardoini* Canzoneri, 1961 and *O. laurae* Canzoneri, 1961. The morphology of the specimens corresponds to that described for *O. laurae*, so we choose to restore this taxon to the species category (see Table 1).

We could not obtain samples from Sicily and Sardinia, and therefore we cannot rule out an independent specific or subspecific status for these populations, either related to *O. laurae* or to *O. unicolor*. Since we cannot make an informed decision on the status of *O. glaber* and its subspecies, nor on *O. u. ardoini*, *O. u. moltonii*, *O. u. chilivanii*, and *O. u. sardous*, we prefer to retain those populations with the taxonomic assignments made by Canzoneri (1961) or Soldati and Soldati (2000).

Thus, considering the current taxonomic results, the partially updated checklist of the genus *Oochrotus* remains as follows: *Oochrotus glaber glaber* Demaison, 1905 (= *O. g. boyadjiani* Lokay, 1907); *O. glaber rhodicus* Koch, 1935; *O. laurae* Canzoneri, 1961, stat. rev.; *O. lusitanicus* Canzoneri, 1961, stat. nov. (= *O. u. espagnoli* Canzoneri, 1961, syn. nov.; = *O. u. hispanus* Canzoneri, 1961, syn. nov.; = *O. u. meridionalis* Canzoneri, 1961, syn. nov.); *O. unicolor ardoini* Canzoneri, 1961; *O. unicolor moltonii* Canzoneri, 1961; *O. unicolor unicolor* Lucas, 1852 (= *O. u. chilivanii* Rallo, 1974; = *O. u. sardous* Canzoneri, 1961).

## ﻿Discussion

The new taxonomic proposal for the genus *Oochrotus* contrasts with both that defined by Canzoneri (1961) and that proposed by Soldati and Soldati (2000). The diversity observed within the genus is not sufficient to define as many taxa as proposed by Canzoneri (1961), but neither is it so homogeneous as to consider all the populations from the Iberian Peninsula, Italy, and North Africa as part of a single species as proposed by Soldati and Soldati (2000). Thus, previous morphological evaluations were unsuccessful in characterising the diversity within the genus, and consequently we are likely dealing with a case of pseudocryptic species, i.e., species that could be differentiated by a very thorough morphological examination but are so similar that there is a high probability of misidentification (Mann and Evans 2008). These species are usually not well identified or remain cryptic until a combined review of morphology and other data, such as ecological, behavioural and/or genetic data, is carried out (e.g. Škaloud et al. 2012; Lajus et al. 2015; Kambestad et al. 2017). In the case of *Oochrotus*, an exhaustive revision of its morphology would be necessary in the future, especially based on Canzoneri’s (1961) descriptions, to define the differentiating morphological traits between species.

There is little fossil information for the entire subfamily Diaperinae, making it difficult to date the divergence processes observed within the genus (Nabozhenko 2019). The unclear phylogenetic proximity between the Iberian or Italian and Moroccan populations would fit with an ancient diversification process as already documented for other tenebrionid beetles (Condamine et al. 2013; Mas‐Peinado et al. 2018, 2022). However, the observed intraspecific diversification pattern supports a possible Pleistocene differentiation, a period known for rapid diversification through the Holarctic region (Knowles 2000; Ribera and Vogler 2004). The phylogeographic structuring observed using the *cytb* marker could point to the beginning of a possible incipient speciation process within refugia formed in the Iberian Peninsula, in a phenomenon widely described for numerous animal and plant taxa and known as “refugia within refugia” (Gómez and Lunt 2007). However, this process did not cause a permanent isolation between populations, since there has been homogenising gene flow between them, as shown by the phylogeography of the highly variable nuclear marker ITS2, a pattern also documented for other Iberian tenebrionids (Mas‐Peinado et al. 2022).

An uncommon evolutionary phenomenon is the presence of multiple base assignment possibilities for single positions in the *cytb* sequence. In our study, individual jgv22035d from Morocco showed alleles common to the haplotypes of the Moroccan and Spanish populations. This pattern may reflect the presence of more than a single mitochondrial genome in an individual, a phenomenon known as heteroplasmy (White et al. 2008). In most organisms heteroplasmy tends to revert to homoplasy within a few generations (Parakatselaki and Ladoukakis 2021). However, in insects heteroplasmic variants can reach the germline (Kondo et al. 1990; Sherengul et al. 2006) and be transmitted for more than 500 generations before disappearing (Solignac et al. 1984; Rand and Harrison 1986). In our focal specimen, the observed pattern could be explained by a recent process of mtDNA hybridisation (Mastrantonio et al. 2019) between *O. lusitanicus* and *O. unicolor* that we cannot confirm with our data. Alternatively, the apparent heteroplasmy could have arisen when both populations were still connected, after which there would not have been enough time for the elimination of the heteroplasmic variants. Other interpretations not related to heteroplasmy might also be possible since mitochondria were not isolated during the DNA extraction process. These include the presence of nuclear-encoded mitochondrial pseudogenes (White et al. 2008) or the retention of ancestral haplotypes shared between Iberian and Moroccan populations due to incomplete lineage sorting (Ballard and Whitlock 2004).

Further research is needed to fully understand the evolution of these beetles. One of the most important issues to be studied is the degree of differentiation and relationships among the Italian populations of *Oochrotus*. From this perspective, the populations studied by Canzoneri (1961) and Rallo (1974) in Sicily and Sardinia are particularly interesting, since islands are systems that favour isolation and speciation (Gillespie and Roderick 2002). Another important point to be studied is the possible involvement of myrmecophily in the evolution of *Oochrotus*. Interspecific relationships, such as mutualistic ones, can have great effects on the population dynamics of the species involved, which is reflected in their phylogeography (Espíndola et al. 2014). From this perspective, it would be interesting to carry out phylogeographic studies on the populations of ants of the genus *Messor*, to study to what extent their patterns are concordant with those of *Oochrotus* and what has been the mutual influence on the evolution of both taxa.

## ﻿Additional information

### Use of AI

No use of AI was reported.

### Author ORCIDs

Julene Gómez-Vicioso https://orcid.org/0009-0004-7043-6579

Álvaro Conca-Esquembre https://orcid.org/0000-0002-9883-4622

Pilar Jurado-Angulo https://orcid.org/0000-0003-1144-9076

Mario García-París https://orcid.org/0000-0002-9361-9405

### Data availability

The data that support the findings of this study are freely accessible in GenBank (see Table 2)
